# Understanding excessive sleep in people with psychotic disorders

**DOI:** 10.1111/bjc.12538

**Published:** 2025-04-02

**Authors:** Kate Robbins, Joanne Hodgekins, Sarah Reeve

**Affiliations:** ^1^ Norwich Medical School University of East Anglia Norwich UK; ^2^ Cambridgeshire and Peterborough NHS Foundation Trust Cambridgeshire UK

**Keywords:** antipsychotic medication, excessive daytime sleepiness, extended sleep duration, functioning, hypersomnia, psychotic disorders, sedation

## Abstract

**Background:**

There has been increasing attention to sleep disturbances such as insomnia in psychosis, due to its impact on symptoms, well‐being, and recovery. However, excessive sleep and extended sleep duration are common in psychosis (partly linked to sedating antipsychotic medication) and have been relatively neglected, despite plausible interactions with symptoms, functioning, and broader well‐being.

**Aim:**

This study aimed to explore the experience of extended sleep duration and excessive sleepiness, or their combination (hypersomnia) in people with psychotic disorders through a qualitative interview around the experience, impacts, contributors, and role of treatment.

**Method:**

Ten patients experiencing excessive sleep (defined as excessive daytime sleepiness >3 days a week; extended sleep duration of > 11 h in 24 h or >9 h at night; or a combination of these) alongside a diagnosed psychotic disorder were recruited. They met with the researcher online to participate in a semi‐structured interview, which was analysed using thematic analysis.

**Results:**

Five major themes were developed: (1) The Exhausting Everyday, (2) Medication is the story? (3) Indescribable Tiredness, (4) Overruled by Sleep and (5) An Unfair Fight. Excessive sleep impacts multiple domains of individual well‐being and recovery – for example, limiting patients in everyday tasks and socializing. Cycles of emotional avoidance and inactivity were identified as potential maintainers or exacerbators of excessive sleep, in addition to medication side effects. Patients reported difficulty conveying the impact of their sleepiness symptoms to clinicians or others.

**Conclusion:**

The results support that excessive sleep requires further attention as a problematic and impactful sleep presentation in this group. Further research is needed to improve recognition and assessment of problematic excessive sleep, and how existing practices or novel treatments may be applied to reduce its impact on recovery.

## INTRODUCTION

Sleep disturbances are common amongst patients with psychotic disorders, with up to 80% found to have symptoms of sleep disorders (Reeve et al., 2019; Waite et al., 2020). The primary focus of work related to sleep disorders has been on sleep‐limiting disorders (e.g. insomnia). Disorders of extended sleep (e.g. excessive sleep/extended sleep duration) have been overlooked and ascribed to the sedating side effects of antipsychotic medications. Initial findings suggest that (1) excessive sleep significantly impacts individual functioning and emotional well‐being, (2) excessive sleep may be influenced by factors beyond medication alone – despite this, there has been a lack of comprehensive studies examining these effects in detail (Faulkner & Bee, 2017; Reeve et al., 2019).

Excessive sleep is here used as an umbrella term, comprising symptoms of excessive sleepiness, extended sleep duration and hypersomnia. Excessive sleepiness can be defined as a ‘propensity to fall asleep when a conscious effort may be needed to stay awake’. Extended sleep duration refers to sleeping more than 11 h in a 24‐h period (including naps) or more than 9 h a night, based on ICSD3 and DSM5 thresholds respectively (ICSD3, American Academy of Sleep Medicine, 2014; DSM‐5, American Psychiatric Association, 2013). Hypersomnia is the combination of excessive sleepiness and extended sleep duration.

Excessive sleep is relatively common in patients with psychotic disorders. A recent study revealed that 23.3% of these patients screened positive for excessive daytime sleepiness during diagnostic interviews (Reeve et al., 2019), and it is frequently reported by clincians (e.g. by over 70% of respondents in Rehman et al., 2016). Often, excessive sleepiness is accepted as a side effect of antipsychotic medications, leading to its perception as an inevitable accompaniment of such treatments (Faulkner & Bee, 2017). However, preliminary findings indicate that factors beyond medication might contribute to the sedation experienced by those on antipsychotics. One study found no differences in medication type or dosage between patients with and without excessive daytime sleepiness (Reeve et al., 2021), and another found that medication changes did not significantly reduce sleep duration (Ramos‐Perdigues et al., 2016). Patients experiencing excessive sleep reported higher levels of depression, anxiety and reduced quality of life, which, despite not being statistically significant, underscores the need for further investigation into the causes of these experiences (Reeve et al., 2021).

There is a critical gap in understanding how hypersomnia symptoms are experienced and interpreted by those with psychotic disorders. To date, no specific qualitative study has been conducted in this area. Given its prevalence and impact on clinically relevant factors, it is essential to deepen our understanding of the clinical presentation, effects and perceptions of excessive sleep. This study aims to be the first to specifically explore the lived experience of excessive sleepiness, extended sleep duration and their combination (hypersomnia) in psychosis.

### Research questions

The current study aimed to answer the following research question:

*‘How do people with psychotic disorders experience excessive sleep?’*

This comprised several elements, including understanding the symptoms and phenomenological experience of excessive sleep; the impacts of excessive sleep on patients; potential causes and contributors either noted by patients or the interviewer; and health care experiences around the assessment or treatment of excessive sleep.

## METHODS

### Study design

This study was a qualitative interview study based on a critical realist framework. Participants were recruited based on consent to follow up from a cross‐sectional survey (on sleepiness in psychosis) and fulfilment of excessive sleep criteria. Data were collected via semi‐structured interviews with people experiencing excessive sleepiness, extended sleep duration, or their combination (hypersomnia) in the context of a psychotic disorder. The data were analysed through thematic analysis using Braun and Clarke's framework (Braun & Clarke, 2006).

### Participants

Participants were recruited via an online survey on sleepiness in psychosis within Cambridgeshire and Peterborough NHS Foundation Trust and on online social media platforms (primarily Facebook and Twitter/X) from April to December 2023.

- Inclusion criteria for completing the online survey were as follows:

- Aged between 18 and 65,
- Fluency in English,
- Diagnosis of first‐episode psychosis (FEP) or a schizophrenia spectrum disorder.

- Exclusion criteria comprised a diagnosis of a mood disorder or a learning disability.

Within the online survey, participants were offered the opportunity to take part in a follow‐up interview which formed the data collection for this study. The interview aspect of the study required participants to fulfil the criteria of excessive sleepiness or extended sleep duration or their combination (hypersomnia), in addition to the inclusion criteria of the survey. Participants were invited if they self‐reported fulfilling at least one of the below additional criteria based on ICSD‐3, DSM‐5, and previous literature (Reeve et al., 2021):

- Reporting ‘feeling excessively sleepy during the day to the extent it was difficult to stay awake or take part in activities’, 3 or more days a week.
- Reporting sleeping ≥11 h within a 24‐h period through self‐reporting yes or no.
- Reporting sleeping ≥9 h at night over the last month, through self‐reporting the number of hours slept.

Eligible participants were emailed the interview information sheet and consent form for the interview. Once consent had been gained, the researcher emailed the participant the topic guide and arranged a time for the interview. Participants were compensated with shopping vouchers for their time and effort in participating in the interviews.

Ten participants were included in the study (Table 1). The majority of participants were female (*n* = 6, 60%), of white ethnicity (*n* = 9, 90%). Ages ranged between 19 and 47 years. The mean age of participants was 43.1 years (SD = 11.09).

**TABLE 1:** Participant demographic characteristics.

Characteristics^a^	*N* (%)
Gender	
Male	4 (40)
Female	6 (60)
Age	
18–25	1 (10)
26–40	5 (50)
41–50	4 (40)
Ethnicity	
White (any background)	9 (90)
Black (any background)	1 (10)
Employment	
Full‐time employed	4 (40)
Part‐time employed	2 (20)
Student	1 (10)
Unemployed	3 (30)
Referral route	
EIP	6 (60)
CMHT	2 (20)
Social media	2 (20)
Diagnosis	
FEP	3 (30)
Schizophrenia	5 (50)
Schizoaffective disorder	1 (10)
Psychotic disorder NOS	1 (10)
Medication	
Antipsychotic and antidepressant	5 (50)
Antipsychotic only	2 (20)
Antidepressant only	1 (10)
Did not specify^b^	2 (20)
Excessive sleep criteria met	
Hypersomnia (≥9 h nightly or ≥ 11 h in 24 h sleep duration, and excessive sleep ≥3 days/week)	6 (60)
Extended sleep only (≥9 h nightly or ≥ 11 h in 24 h sleep duration)	2 (20)
Excessive sleep only (≥3 days/week)	2 (20)

Abbreviations: CMHT, community mental health team; EIP, early intervention in psychosis; FEP, first episode psychosis.

^a^ All participants demographic information was self‐reported.

^b^ All participants confirmed they were taking psychiatric medication, specifying type and dosage was optional.

There were a range of diagnoses in the group including schizophrenia (*n* = 5, 50%), first‐episode psychosis (*n* = 3, 30%), with the remaining two participants each diagnosed with schizoaffective disorder and psychotic disorder not otherwise specified. All participants reported taking psychiatric medication, although *n* = 2 (20%) did not specify which medication they were prescribed. Of the remainder, five (50%) reported taking an antipsychotic and an antidepressant, two more were prescribed an antipsychotic only, and one was prescribed an antidepressant only. Hypersomnia (the combination of excessive sleepiness and extended sleep duration) was experienced by 60% of the participants. Excessive sleepiness was experienced by 20% of the participants. Extended sleep duration was experienced by 20% of the participants.

### Interview design

The topic guide (see Appendix S1) included questions that were designed to answer the research question and map on to the domains of the experience, impact, contributors, role of medication, and treatment of excessive sleepiness. Within each broad domain, prompting questions were listed in the topic guide to cover subdomains, if needed. At the end of the interview, participants were invited to return to any of the questions answered or discuss any topic related to their excessive sleepiness that was important to them, which had not been asked in the preceding questions.

The study process and topic guide were reviewed by a small sample of lived experience representatives, in line with Public and Participant Involvement (PPI) procedures. The group made suggestions on terminology, specifically to use ‘excessive sleepiness’ within the interviews to utilize lay language; the topic guide was adjusted to reflect this advice. PPI also advised that the topic guide was emailed to participants in advance of the interview to facilitate the discussion, which was incorporated into the study protocol.

All interviews were conducted online via Microsoft Teams and lasted between 30 min and 1 h. The interviews were transcribed using transcribing software within Microsoft Teams and then reviewed for accuracy against the recording before being uploaded to NVivo for coding QSR International (2017) NVivo (Version 12). The video recording was subsequently deleted, with the written transcript being the sole unit for analysis.

### Analysis

Inductive thematic analysis, as defined by Braun and Clarke (2006), emphasizes a bottom‐up approach where themes are derived directly from the data, rather than imposing preconceived categories or theories. This approach was chosen due to the study's aims to explore the lived experiences of participants, and in order to provide a framework to account for bias arising from the researchers' roles as clinicians and researchers.

The first step of the analysis involved familiarization with the data by reviewing the transcripts, carried out by KR as the primary researcher. During this phase, a reflexive journal was utilized to support in maintaining a broad approach to the data and noting initial thoughts, recurring ideas, phrases and concepts. Consultation within a research peer qualitative forum and research supervision with SR and JH aided this process. Initial coding was then undertaken by KR within NVivo of all transcripts. Codes were grouped into initial themes by KR, with this process reviewed and discussed with SR, including reflexive discussion of researcher positioning in relation to the topic. Following subsequent iterations, the final theme framework and names were agreed upon by all authors.

### Ethical considerations

This study was approved by an NHS Research Ethics Committee (ref: 23/LO/0085) and was sponsored by the University of East Anglia.

## RESULTS

**FIGURE 1:**
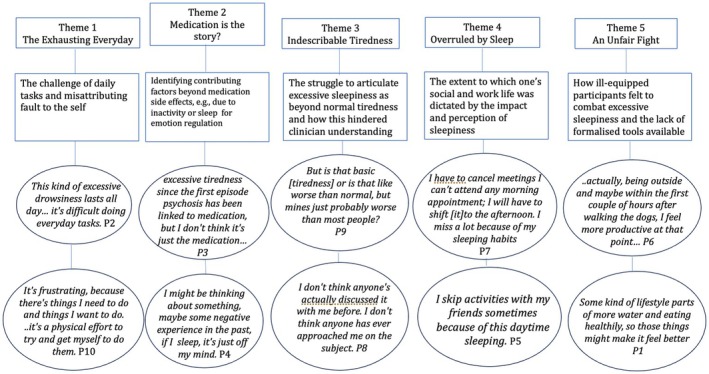
Presentation of thematic analysis themes, corresponding descriptions and quotes.

From the thematic analysis, five themes were developed as follows: (1) The Exhausting Everyday, (2) Medication is only a part of the story, (3) Indescribable Tiredness, (4) Overruled by Sleep and (5) An Unfair Fight. A diagram of the themes and their descriptions, alongside key quotes, is presented in Figure 1.

### Theme 1: The exhausting everyday

The challenge of everyday tasks was a point emphasized by many of the participants. Participants emphasized how tasks such as shopping or housekeeping would feel momentous in the face of sleepiness, as the exhaustion and time consumption would result in a feeling of lost time during the day:

I had to deal with the disappointment of missing hours in the day. P9

The impact of not being able to achieve the day‐to‐day tasks considered straightforward by others resulted in a detrimental impact on participants emotional well‐being. Frustration and self‐criticism were evident, and some participants spoke emphatically about feeling they were not reaching their full potential and feelings of sadness and worry around this:

I feel like I've got missing potential that other people have, so don't feel like I'm good enough. P8

I would say that it makes me feel down because you [are] constantly taking longer to do stuff …where some people would do three or four tasks and you compare it to your one. P9

I feel very worried. Because of the things that I am supposed to do; I don't feel able to do them. P5

Participants would often attribute the responsibility of not being able to do enough during the day to their personal abilities, as opposed to the mental health challenges they were experiencing. This had a consequent impact on participants self‐esteem and feelings of embarrassment. Overall, the private daily battle of not being able to do enough appeared to contribute to a societal comparison where participants would unfairly view themselves as less capable than others and internalize feelings of shame as a result:

I was quite hard on myself; I used to think other people are at work now and I not achieving enough. It has impacted my self‐esteem a bit. P8

### Theme 2: Medication is the story?

Medication was discussed by participants as a contributing factor to their excessive sleep, alongside other factors, such as utilizing sleep to block out emotions and inactivity resulting in increasing feelings of sleepiness:

I think that being on the Olanzapine has made me excessively sleepy as well… but I think that there's an element to the excessive sleepiness that has nothing to do with the Olanzapine… P3

Participants varied in the emphasis that they placed on medication being the cause of excessive sleep. When asked about the cause of their sleepiness, three participants responded directly that they believed it to be due to their medication side effects, in some cases stressing that they were not doing it to themselves.

I think the excessive sleepiness started when I started taking the antipsychotics. P1

I really would say that it's mainly the clozapine it's not like I'm kind of losing sleep in the evening or something, going out clubbing… No, it really is the clozapine. P2

Six of the 10 participants mentioned medication alongside other factors. One participant did not specify the cause of their sleepiness. Emotional well‐being was frequently referenced as a relevant factor. Participants described how anxiety and depression alongside psychotic symptoms would interact with the sleepiness. Feelings of worry or sadness were dealt with by inviting sleep in and utilizing it as a way to escape feelings or re‐set. This was felt to be an effective way to shut off from emotions:

If there's something I'm dreading, then I feel sleepy and I just want to nap until the world feels like a better place… for example, I was worried about a phone call with a friend, and the day before I was due to have that phone call I was feeling very sleepy and I went and had a nap because I was just like, ohh, I can't face it. P3

A cyclical relationship was described by participants where emotional states could also cause feelings of sleepiness:

Because I do a high impact job and the stress of doing the job, having what you suffer with, it makes me more sleepy. P9

Participants described inactivity as another contributing factor to excessive sleep. A cyclical relationship was described whereby sleepiness would impact motivation and result in doing less, which in turn would result in stronger feelings of sleepiness. This cycle could also include sleepiness preventing the ability to engage in a full‐time career, which in turn would result in doing less and feeling sleepier as a result:

I think I often don't do enough during the day and then I'm not doing enough, and then I don't sleep very well, and it just sort of snowballs from there. Feeling sleepy during the day and then not being active enough as a result. I think that can be a sort of negative spiral I can be in. P3

I think because I am unemployed, I don't have something driving me to fight through the tiredness. P8

### Theme 3: Indescribable tiredness

Participants described a pervasive challenge in articulating the reality of excessive sleep to others, leading to feeling of being misunderstood by health care professionals. This challenge was also evident in the interviews – in many cases participants would pause and emphasize tonally how they were feeling, with a felt sense of desperation. Many participants described the tiredness they felt as being beyond ‘normal’ tiredness. For the participants, the sleepiness experienced was beyond comprehension to those who had not had to endure it:

I think everybody has days where they feel tired but that isn't what excessive sleepiness is it's worse than that and it's more pervasive. It was not one night of feeling tired, it's a week or month's worth of feeling tired, where you're just kind of feeling on adrenaline. And you just, maybe I didn't say this quite as well as I could … Like it's almost like you're just existing rather than being the person you could be. P10

In the endeavour to translate the experience of excessive sleep, some participants drew on the physiological aspects. Experiences of brain fog and trouble concentrating were discussed, in addition to a feeling of ‘heaviness’ and being weighed down by sleep:

I'll feel a lot more heavier to carry myself you know. P4

I just feel tired and get this general feeling of brain fog because I'm so tired and lack of focus on what I'm doing. P2

To facilitate an understanding of the daily experience of excessive sleep, participants attempted to relate to common sleep experiences that are more widely recognized. In the example, the participant here uses the allegorical explanation of being woken up in the night and the feeling of disorientation as a result. The sense of wanting to really translate the experience to everyday experiences was felt here, in an attempt to make the unrelatable relatable:

It feels like if there's some reason you're woken up in the middle of the night… sort of sleep inertia where it's hard to wake up… I just really want to go back to sleep. P2

Similarly, to the use of common sleep experiences, participants also tried to explain excessive sleep using similes:

…. it's almost like a form of feeling bad and form of torture. P2

it's like a big heavy blanket on my back. P8

The difficulty in conveying the experience also carried through to medical care, with some participants reporting feeling dismissed or misunderstood in their discussions with health care professionals:

You don't feel listened to. I've mentioned it once, and it was shrugged off a bit. I said to my psychiatrist that I was tired, and basically it was like, yeah, it's just it's getting darker nights and cold weather, and it was a bit like ohh, ok, I don't really wanna talk anymore about that. P9

In contrast, some participants did report health care professionals taking action on their excessive sleep, although in all cases the solutions provided were medication related.

When I went on the olanzapine and then kind of switched over to the aripiprazole. So, I was with a really good psychiatrist who's really helpful, was very understanding of what I was going through was not dismissive. P10

No participants reported discussions with health care professionals on excessive sleep outside of a medication context.

### Theme 4: Overruled by sleep

Participants reported their lives being dictated by sleep, in planning time around sleeping, cancelling plans, and missing out on work opportunities. The greatest impact was felt in participants' social lives where they described feelings of detachment and self‐blame for prioritizing rest:

Basically, I kind of have very few relationships, so I think that's kind of me being more insular because of my schizophrenia and my excessive sleepiness. I don't have any friends due to the excess sleepiness, which prevented me forming social contact. P2

Participants described the extent to which they had to schedule their time around either the hours of sleep they needed or the levels of sleepiness they expected themselves to feel. For some, this meant scheduling all their important appointments for the afternoons; for others, adjustments were more ad hoc due to the unpredictability of how sleepy they might feel:

…and in the times when I'm really struggling with morning sleepiness and struggling to get up at a sensible time, I arrange everything for the afternoon basically. P3

As a result of the sleep needs of the participants, some reported missing out on job opportunities, promotions or having a job at all. Participants described a sense of loss at not being able to fulfill their potential or take opportunities. A cyclical nature to work and sleepiness was discussed in feeling fearful of how one would manage in a job due to the added stress of needing to adjust their work to their sleep needs or having to discuss this with a manager. For one participant, the amount of sleep needed had resulted in the termination of a previous contract and present unemployment:

Obviously, I don't work at the moment. And that's a big concern of mine, because I want to try and get back into work. But I'm very conscious of how that what that will look like if I'm having a bad day. And I previously lost a job. Because, you know, the sleepiness and the illness was so bad that I couldn't work at all. And that's a real worry for me as well is re‐joining the workforce and what that looks like. P10

Outside of the realm of employment and education, the dominance of needing to sleep had a significant impact on participants ability to engage socially. Participants reported how extreme tiredness, especially in the evenings, would result in missing out on social plans due to a need to rest:

…and I think there have also been times when I've struggled to sort of meet up with friends in the evenings because I would be getting very sleepy in the evening as well… there have been times when it's really interfered with my social life. P3

Participant accounts reflected self‐criticism on missing social opportunities due to sleepiness. A fear of what others might think of sleepiness being a reason for absence was highlighted. Once more, in the challenge of explaining sleepiness outside of normal tiredness, there was a worry that one would be misunderstood and feel to be giving a poor excuse for social disengagement. As a result, a cycle of avoidance could occur, where social interactions would be avoided due to fear of judgement:

I don't know if they think that I'm being lazy… or that I should just try and push through it. More that isn't a sufficient reason not to [socialise]. P10

I don't want to be one of these people that just goes out with their friends and complain about being tired or being exhausted. I just stay at home and have a cup of tea. P6

Finally, participants lamented how sleepiness had resulted in a withdrawal of activities previously enjoyed. Accounting for this, participants cited the general experience of sleepiness as impactful on the motivation to do recreational activities:

And then also I've been struggling to go to as many activities as I used to do… I don't do as much ballet or as many cool things as I used to… P1

### Theme 5: An unfair fight

Participants felt outmatched by their sleepiness and had few tools to combat it. Where resources were drawn upon, participants referred to tactics that they had learnt themselves, which mapped on to basic advice for sustaining well‐being, such as getting fresh air or drinking water:

When I'm first up and I push myself to walk the dogs. And I actually feel quite energised during a dog walk and afterwards. P6

… getting out actually helps. So, if I'm really, really, really tired, sometimes the act of physically pulling myself out house and having some fresh air can kind of give me a little bit of energy. P10

The language used by participants in describing ways they sought to remedy excessive sleep was illustrative of the challenge they felt. Many participants referred to ‘fighting’ the sleepiness through engaging in activities that were beneficial to their mental health, such as getting fresh air, exercise or going for a walk. However, these endeavours were impeded by the physiological experience of excessive sleep. A sense of needing to force oneself into action was explained when descriptions of fighting the sleepiness were discussed:

…. I think it's actually countering the lack of focus, lack of motivation which comes from excessive tiredness…. It kind of helps if you can force yourself into doing something even if you don't quite feel like doing it. P3

Participants largely drew on self‐help tactics, such as distraction when describing how they may combat sleepiness. Avoiding napping was cited as helpful, although challenging. A number of participants described drinking coffee or caffeinated drinks to combat sleepiness, although with potential side effects of increasing anxiety:

But I do fight it like I've said before, you know, I've got my distraction techniques which, you know, hopefully are helping me. And sometimes when reading newspaper, I make a special effort to kind of read every article in it, instead of kind of letting my tiredness stop me from reading it altogether. P2

caffeine helps me not feel as tired. But the flip side of that is that it makes my anxiety worse, the more caffeine I have. P10

In two cases, participants described having taken specific therapeutic techniques and adapted them to tackle their sleepiness. Psychoeducation was felt to be beneficial in normalizing the experience of sleep inertia on waking:

[the psychologist] recommended some handouts on sleep hygiene… talked about sleep inertia, which is a really helpful context. So basically, when I get up in the morning and I'm feeling like ohh, I can't face it like I'm clearly too sleepy to get up and like, no, this is just sleep inertia. P3

One of the most effective tactics described by a participant was a simple task of goal setting within cognitive behavioural therapy (CBT). The process of having a therapeutic goal that was geared towards skills building whilst taking into account the sleepiness was described positively. The therapeutic accountability was also deemed to be helpful, and the positive sense of achievement afterwards helped to counter the aforementioned feelings of damaged self‐esteem at not being able to achieve enough:

[In] CBT [the therapist] did try say you should try and achieve things. For instance, I wanted to learn how to cook, and I made gains in that area, … the important thing is you set yourself these tasks every week and you then achieve them. You kind of do them even if you're really tired, or if you're motivation is really low and you feel you can't. It forces you to kind of get that focus back. And I would say that is certainly something that's helping with regards to excessive sleepiness. P2

Although the relationship between sleep and hearing voices was mentioned infrequently, one participant described how sleepiness made using therapeutic techniques such as CBT‐based evidence evaluation more challenging:

Because the sleepier I am, the harder it is for me to say to myself or to the voices, what evidence is there to support that? I mean, I sleep, because I'm so exhausted. It's just it dreamless sleep. And so, it's just everything else goes away. P10

## DISCUSSION

This study was the first examination of the experience of excessive sleep in people with psychotic disorders. The findings of the study are threefold. Firstly, excessive sleep is a broad phenomenon that affects a range of domains in people's lives. Secondly, cycles of behaviour, such as avoidance and inactivity, may contribute to the presence of excessive sleep, in addition to medication being a clearly attributed cause of sleepiness. Thirdly, excessive sleepiness is challenging to articulate at an individual level, which limits the ability for it to be assessed or addressed in clinical services and consequently, patients are often left to manage these chronic difficulties on their own.

Excessive sleep is impactful on daily living. As a result of excessive sleep, people can feel inadequate in what they are able to achieve during the day, leading to an anxiety of not doing enough, frustration, and a sadness of lost potential. The impact of sleepiness has indications for the ability to participate in the workplace and to socialize effectively. These are important factors which have indications for contributing to depression, anxiety and lowered quality of life. The recovery model approach for psychotic disorders incorporates an examination of factors beyond a reduction of symptomology alone (Leonhardt, 2020). Social recovery is a high priority for patients; it is possible that excessive sleep might be a contributor to limited social recovery in psychosis and warrants more explicit consideration (Fowler et al., 2019; Hodgekins et al., 2015).

Cycles of avoidance and inactivity contributed to the maintenance of excessive sleep – beyond medication alone. The results support previous research which highlighted that excessive sleep is not solely explained through the sedating side effects of antipsychotic medication (Reeve et al., 2021). What has not been previously known is what is contributing to excessive sleep at an individual level. This study has evidenced that people may be unintentionally contributing to excessive sleep by utilizing sleep as a coping strategy for difficult emotions or becoming stuck in a cycle of inactivity caused by the impacts of sleepiness. Existing treatments, such as CBT could target these maintenance cycles, as evidenced within this study where CBT approaches enabled one participant to feel increases in personal productivity and consequent improvements in their sleepiness.

Participant's locating the responsibility of sleepiness to the self is worth further consideration. Through comparison with others, and with a lack of clarity of the complexity of the potential causes of sleepiness, participant's self‐esteem and self‐efficacy were compromised. A negative appraisal of the responsibility of the presence of sleepiness to the self contributed to cycles of inadequacy and self‐doubt, which in turn impacted individual mood and perceptions of self‐worth. Such beliefs could be targets for further intervention, in line with existing CBT models and patient preferences (Freeman et al., 2019).

Assessment and measurement of sedation and excessive sleep is a key challenge. Previous studies have highlighted the lack of uniformity in measuring the sedating side effects of antipsychotic medications (Longden & Read, 2016). This study has highlighted how an individual challenge of articulating the experience may have also contributed to its current clinical conceptualization, converging with evidence that sedation concerns are not well‐addressed in clinical consultations, potentially in comparison to other side‐effects (Seale et al., 2007). Whilst there are some excessive sleep and hypersomnia measures – e.g. Epworth Sleepiness Scale and Hypersomnia Severity Index (ESS; Johns, 1991; Kaplan et al., 2019) the former is not suitable for psychosis as it requires exposure to public places, and the latter is not widely used. The results indicate that a more comprehensive examination of the individual impacts of excessive sleep is needed. The development of a suitable patient‐reported tool, for example, could facilitate clinical communication around excessive sleep and inform the management of sedation, capturing the severity of the experience to aid in decision on prescribing and therapeutic inputs.

### Clinical implications and recommendations

The development of a specific validated tool which can measure/explore excessive sleep in line with its functional impacts and relationship with emotional regulation is needed. An exploration of excessive sleep should be carried out at assessment and at key points within treatment to map how an individual may be relating to their sleepiness. Following a sufficient measurement of excessive sleepiness, potential patterns related to sleepiness should be considered as targets within therapy. Existing treatments such as behavioural activation, psychoeducation on sleep and its interactions with medication and mental well‐being, and goal setting should be considered. Individual daily functioning should be assessed in relation to their sleepiness to highlight where unemployment or lack of social support may be contributing to or impacted by sleepiness.

### Limitations and future research

The sample in the study is drawn from people experiencing excessive sleep in the context of a psychotic disorder, and who responded to an online survey around this topic. The study sample is predominantly female, employed, and white, and therefore less representative of the wider population of those with psychosis. Future research should aim to explore the experiences of a wider range of people with psychotic disorders to continue to enhance the understanding of excessive sleep. This is particularly important for examining the interaction between demographic factors such as gender and ethnicity on prescribing practices and recovery (Morgan et al., 2017; Wang et al., 2023), and the potential impact of excessive sleep on employment for those who may already be socially disadvantaged.

This study did not screen participants on the type of antipsychotic medication taken, specific psychotic disorder, or duration of treatment. Future studies could recruit participants based on medication type or record changes in the relationship to antipsychotic medication over time. This study used self‐report to screen for excessive sleep and sleepiness and did not use a validated measure for screening purposes. Future studies could incorporate objective measures of sleep and sleepiness (e.g. actigraphic monitoring).

With respect to the analysis, the primary coding and review of transcripts was carried out by one researcher (KR), which may have introduced bias or error into the process. This was mitigated by the use of research supervision and peer supervision, and by the use of a reflexive log throughout the process of coding and analysis. Future studies could utilize lived experience researchers or those with other perspectives (e.g. non‐psychologist clinicians) within the analysis process to generate further meanings and thematic interpretations for further research.

The study focused on the experience of people experiencing excessive sleep in the context of a psychotic disorder and was therefore limited to this perspective and to the approach of inductive thematic analysis. Future research could explore the perspectives of other individuals such as carers or clinicians or utilize alternative approaches such as grounded theory in order to build a more robust theoretical model of how excessive sleep affects individuals with psychosis.

## CONCLUSION

This study is the first to qualitatively explore excessive sleep in people with psychotic disorders. Findings indicate that excessive sleep impacts daily functioning, emotional well‐being, and social relationships. It is influenced by inactivity and used as an emotion regulation strategy, in addition to being a medication side effect. This complex relationship requires thorough, individualized assessment. Such assessments are crucial for identifying the contributors to excessive sleepiness, leading to personalized and effective management. Recognizing its impact can validate patients' experiences, fostering a supportive clinical environment. Improved assessments can inform both existing and new treatment approaches, with the potential to enhance overall well‐being and quality of life for those affected.

## AUTHOR CONTRIBUTIONS

**Kate Robbins:** Conceptualization; investigation; methodology; formal analysis; writing – original draft; writing – review and editing. **Joanne Hodgekins:** Conceptualization; supervision; writing – review and editing; methodology; formal analysis; project administration. **Sarah Reeve:** Conceptualization; methodology; supervision; writing – review and editing; formal analysis; project administration.

## CONFLICT OF INTEREST STATEMENT

No relevant conflicts of interests to declare in relation to this article.

## Supporting information


Appendix S1.

## Data Availability

The data that support the findings of this study are available on request from the corresponding author. The data are not publicly available due to privacy or ethical restrictions.
