# Supplementary material for: Understanding excessive sleep in people with psychotic disorders
Source: Br J Clin Psychol. 2025 Apr 2;65(3):762–76. doi: 10.1111/bjc.12538 (PMC13456434; doi:10.1111/bjc.12538)
Supplement: Supplementary file 1 [file BJC-65-762-s001.docx]

**Interview Topic Guide**

**Topic 1: Experiences of excessive sleepiness**

*Opening statement: Thank you for coming today, I really appreciate you taking the time to meet with me. I am interested in your experience of excessive sleepiness.*

*I would really like to learn what this experience is like for you and I will start by asking you about your experience of excessive sleepiness. Following this, I will have some questions to gain more detail on what you have told me* (these will be guided by what they described)*. What is important to me is to really understand your experience, nothing is irrelevant, I’m interested in what this experience is like for you. You don’t have to answer any questions that you don’t want to answer. I will try to listen as much as possible and not interrupt you, but if you have any questions as we go along please do ask me.*

*Please tell me about your experiences of excessive sleepiness?*

**Topic 2: Impact of excessive sleepiness**

*What is it like to experience excessive sleepiness ?*

*What impact does experiencing excessive sleepiness have on you?*

- *Prompts, explore impact on:*
  - *Day to day living*
  - *Wellbeing*
  - *Relationships*
  - *Treatment*

**Topic 3: What may cause or contribute to excessive sleepiness**

*What do you think could be the cause of the excessive sleepiness that you experience?*

*Is there anything that you do that can make it better?*

*Is there anything that you do that can make it worse?*

**Topic 4: Role/Experience of medication**

*What role does your medication have in your experience of excessive sleepiness?*

**Topic 5: What helps with excessive sleepiness**

*What do you find helpful in your experience of excessive sleepiness?*

**Topic 6: Ending the session**

*Is there anything that I haven’t asked / we haven’t discussed about excessive sleepiness that you think is important for health professionals to know?*

*Is there anything you would like to talk about that we haven't talked about?*

*Closing statement: Thank you so much for spending the time with me today to discuss your experience of excessive sleepiness. We will be in contact via email with a £20 voucher to thank you for your time.*
